# A role for intestinal alkaline phosphatase in preventing liver fibrosis

**DOI:** 10.7150/thno.48468

**Published:** 2021-01-01

**Authors:** Yang Liu, Paul M. Cavallaro, Byeong-Moo Kim, Tao Liu, Hongyan Wang, Florian Kühn, Fatemeh Adiliaghdam, Enyu Liu, Robin Vasan, Ehsan Samarbafzadeh, Matthew Z. Farber, Junhui Li, Meng Xu, Vidisha Mohad, Michael Choi, Richard A. Hodin

**Affiliations:** 1 Department of General Surgery, Xi'an Jiaotong University Second Affiliated Hospital, Xi'an, China.; 2 Department of Surgery, Massachusetts General Hospital, Harvard Medical School, Boston, MA, US.; 3 Division of Gastroenterology, Massachusetts General Hospital, Harvard Medical School, Boston, MA, US.; 4 Department of Gastroenterological Surgery, First Affiliated Hospital of Zhengzhou University, Zhengzhou, China.; 5 Department of Gastroenterological Surgery, People's Hospital of Liaoning Province, Shenyang, China.; 6 Department of General, Visceral, and Transplant Surgery, Ludwig-Maximilians-University Munich, Munich, Germany.; 7 Department of Hepatobiliary Surgery, Qilu Hospital of Shandong University, Jian, China.; 8 Department of Surgery, University-Pittsburgh Medical Center, Pittsburgh, PA, US.

**Keywords:** Liver fibrosis, intestinal alkaline phosphatase, gut barrier, TLR4

## Abstract

**Rationale:** Liver fibrosis is frequently associated with gut barrier dysfunction, and the lipopolysaccharides (LPS) -TLR4 pathway is common to the development of both. Intestinal alkaline phosphatase (IAP) has the ability to detoxify LPS, as well as maintain intestinal tight junction proteins and gut barrier integrity. Therefore, we hypothesized that IAP may function as a novel therapy to prevent liver fibrosis.

**Methods:** Stool IAP activity from cirrhotic patients were determined. Common bile duct ligation (CBDL) and Carbon Tetrachloride-4 (CCl4)-induced liver fibrosis models were used in WT, IAP knockout (KO), and TLR4 KO mice supplemented with or without exogenous IAP in their drinking water. The gut barrier function and liver fibrosis markers were tested.

**Results:** Human stool IAP activity was decreased in the setting of liver cirrhosis. In mice, IAP activity and genes expression decreased after CBDL and CCl4 exposure. Intestinal tight junction related genes and gut barrier function were impaired in both models of liver fibrosis. Oral IAP supplementation attenuated the decrease in small intestine tight junction protein gene expression and gut barrier function. Liver fibrosis markers were significantly higher in IAP KO compared to WT mice in both models, while oral IAP rescued liver fibrosis in both WT and IAP KO mice. In contrast, IAP supplementation did not attenuate fibrosis in TLR4 KO mice in either model.

**Conclusions:** Endogenous IAP is decreased during liver fibrosis, perhaps contributing to the gut barrier dysfunction and worsening fibrosis. Oral IAP protects the gut barrier and further prevents the development of liver fibrosis via a TLR4-mediated mechanism.

## Introduction

Fibrosis is a common sequela in many chronic liver diseases, including hepatitis, benign or malignant bile duct obstruction or alcoholic liver disease. In recent years, an important relationship between liver pathology and the gastrointestinal tract has come to light 1. In particular, the pathophysiologic development of liver fibrosis has been linked to an impaired GI tract barrier. The intestinal barrier sits at the interface between the host and a microbial population that is the source of important mediators of intestinal and hepatic inflammation 2, 3. Previous studies have reported that decreased tight junction protein expression and increased gut permeability occurs during the early stages of both common bile duct ligation (CBDL) and Carbon Tetrachloride-4 (CCl4) induced liver fibrosis murine models. Further, intestinal bacterial overgrowth is often associated with liver fibrosis 4; the increased burden of gut bacteria and pathogen associated molecular patterns (PAMPs) allow for translocation from the gut into the liver parenchyma via the portal venous system 4. Interestingly, the depletion of intestinal bacteria by a cocktail of antibiotics or blockade of the lipopolysaccharides (LPS)-TLR4 pathway rescues liver fibrosis in murine models 5, thereby implicating the importance of the microbiome and microbial products in the development of this disease.

Intestinal alkaline phosphatase (IAP) is one subtype of the alkaline phosphatase family and is produced exclusively in the intestine with highest expression in the duodenum 6. Endogenous IAP levels have been shown to be lower in gut-associated inflammatory conditions such as inflammatory bowel disease and diabetes mellitus 7-9. IAP levels have not been previously assessed in the context of liver fibrosis.

IAP is known to detoxify bacterial LPS by removing a phosphate group from its lipid A moiety, thus preventing recognition by its receptor TLR4 10. In addition, IAP maintains the gut barrier by up-regulating intestinal tight junction protein expression 11. Mice lacking the IAP gene characteristically display an impaired gut barrier and increased intrahepatic inflammatory markers, and these findings become more prevalent with age 12. It has been shown that in a mouse alcohol model that mice lacking IAP display accelerated liver steatosis, whereas oral supplementation with IAP alleviates the development of steatosis 9. Given these observations, we hypothesized that IAP may rescue the liver from the development of fibrosis in other models of hepatic injury, specifically common bile duct ligation and CCl4 toxicity.

## Methods

### Human samples collection

Stool samples were collected from patients with liver cirrhosis hospitalized in the Liver Disease Center at the Second Affiliated Hospital of Xi'an Jiaotong University. Control stool samples were collected from patients diagnosed with inguinal hernia, gallstones, and thyroid nodular goiter and with no cirrhosis seen in the surgical clinic at Department of General Surgery, Second Affiliated Hospital of the Xi'an Jiaotong University before their operation.

### Animals

IAP KO mice (C57BL/6 background) mice were generated by the Sanford-Burnham Medical Research Institute (La Jolla, CA) 13 and TLR4 KO (C57BL/6 background) mice were generously gifted from the Mucosal Immunology and Biology Research Center at Massachusetts General Hospital. IAP KO and TLR4 KO littermates were bred at the specific-pathogen-free (SPF) rooms within the Center for Comparative Medicine (CCM) at the Massachusetts General Hospital (MGH) with 12-hour light-dark cycles. All animal experiments (protocol 2017N000152) were reviewed and approved by the Institutional Animal Care and Use Committee at MGH.

### Oral IAP in drinking water

IAP was dissolved in autoclaved tap water to a final concentration of 200 IU/mL. IAP was given 4 days prior to bile common duct ligation or CCl4 injection and then continued for the remainder of the experiment. The drinking water supplemented with IAP was changed every day.

### IAP activity assay

Stool samples were diluted at a ratio of 1:30 (weight:volume) by stool dilution buffer (10 mM Tris HCl, PH 8.0; 1 mM MgCl_2_; 10 μM ZnCl_2_) then homogenized with glass beads for 10 min. The homogenate was then centrifuged at 10,000 g for 10 min and the supernatant was taken for analysis. Total protein concentration was measured by the Bradford assay. To measure IAP activity, 25 μL or supernatant was added to 175 μL pNpp solution (1 mL 1 M Tris-HCl pH 8.0, 1 M MgCl_2_, 10 mM ZnCl_2_, 186 mg pNpp, 99 mL Water) and OD405 was measured over time. In separate wells, the selective IAP inhibitor phenylalanine (10 mM) was added to the pNpp solution. Bovine IAP (Sigma) was used to create a standard curve. Change in OD405 over time was normalized to total protein concentration for each sample, and the result with phenylalanine was subtracted from the result without phenylalanine. The final result was reported as units IAP/mg protein.

### Statistical analysis

The Prism 7.0 software (GraphPad, San Diego, CA) was used to analyze the data, shown as mean ± SD, and a two-tailed student's *t*-test or one-way analysis of variance was used to compare groups. A *p* value <0.05 was considered as significantly different.

Other detailed methods can be found in the Supplementary Information.

## Results

### Fecal IAP activity decreases in humans with liver cirrhosis

A total of 18 control patients without cirrhosis and 86 patients with liver cirrhosis had stool samples collected. The mean age of liver cirrhosis patients was 48.08 ± 10.16 with 51 males and 35 females. The etiology of liver diseases included hepatitis B virus (n= 53), hepatitis C virus (n= 12), HBV and HCV (n=3), alcoholic cirrhosis (n=6), autoimmune cirrhosis (n=5), cholestatic cirrhosis (n=4), and unknown causes (n=3). The Child-Pugh classifications of these patients were as follows: 27 class A patients, 30 class B patients, and 29 class C patients. The non-cirrhosis control samples were collected from patients with biliary colic (n=8), inguinal hernia (n=6) and thyroid nodular goiter (n=4) with a mean age of 45.39 ± 12.52. The liver function was class A in all control group patients. The characteristics of these patients are shown in **Table 1.**

**Figure 1:**
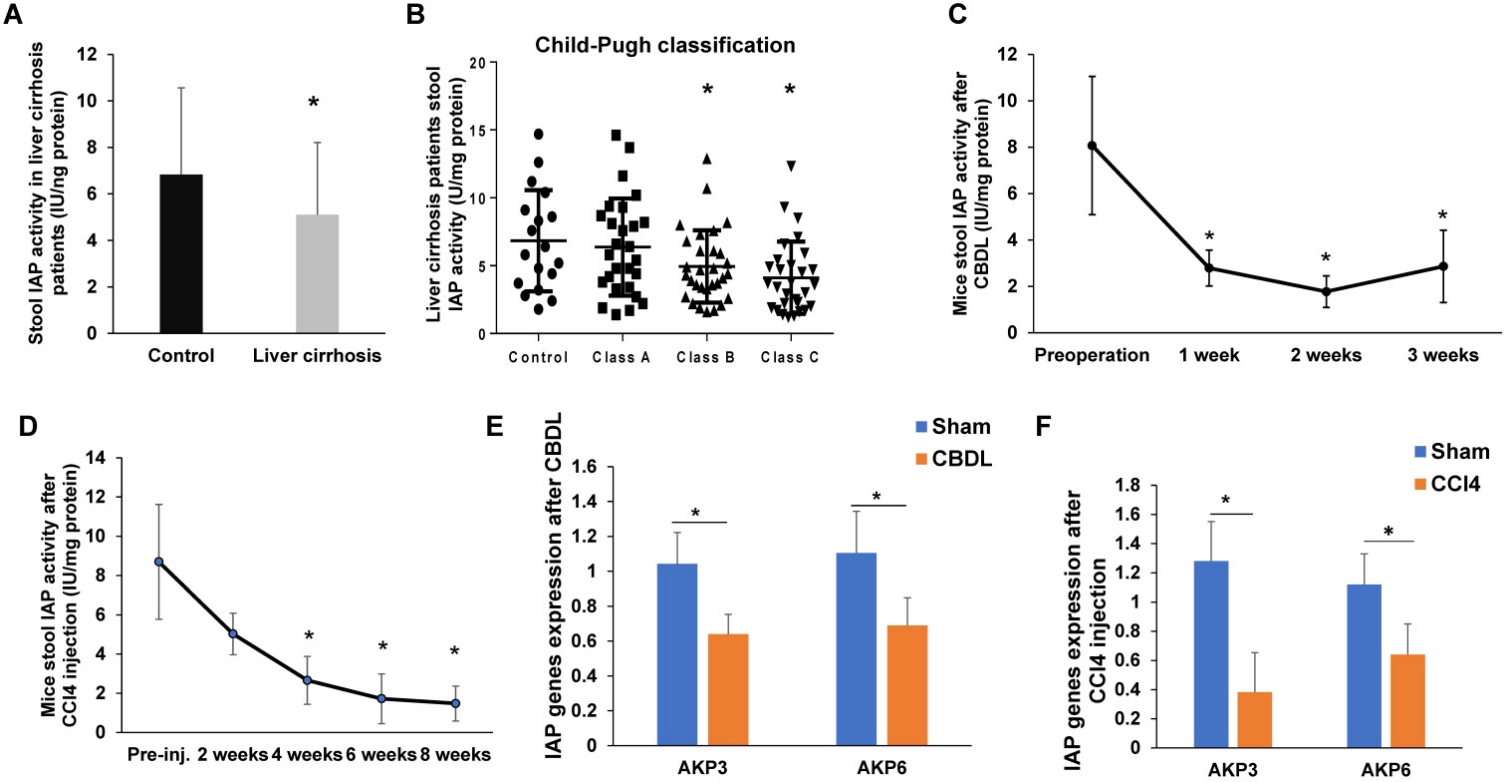
** IAP expression is decreased in liver fibrosis in both mouse and human**. (A) Stool IAP activity from liver cirrhosis patients VS. control measured by pNPP assay; (B) Stool IAP level of liver cirrhosis patients in different liver Child-Pugh classifications measured by pNpp assay; (C-D) Stool IAP activity in CBDL (C) and CCl4 (D) induced liver fibrosis models measured by pNpp assay at different time-points after the procedure. (E-F) Mouse duodenal AKP3 (duodenal IAP) and AKP6 (global IAP gene) gene expression detected by qPCR methods in both models. *: *p*<0.05.

Compared to patients without cirrhosis, patients with cirrhosis had significantly lower fecal IAP activity (*p*<0.05, Figure 1A). When comparing IAP activity by Child-Pugh grades, we found a decreasing trend as liver function worsened. Although, there was no difference in stool IAP activity between control patients and patients with class A liver function, control samples had a significantly higher IAP activity when compared with Child-Pugh B and C patients (*p*<0.05, Figure 1B).

### IAP activity and expression decreases during murine liver fibrosis

Given that liver fibrosis is associated with chronic intestinal inflammation, and endogenous IAP levels have been shown to be decreased in a variety of models of intestinal inflammation 7, 9, 14-16, we first measured stool AP activity using pNPP as a substrate for IAP. For mice that underwent CBDL, stool IAP activity significantly decreased from baseline preoperative stool IAP activity levels by 1 week postoperatively (*p*<0.05), and activity remained reduced when measured at both 2 weeks and 3 weeks after surgery (Figure 1C). Similarly, in mice that received CCl4 injections, stool IAP activity began to decline from baseline after 2 weeks of injections. Stool IAP activity was significantly less than baseline at 4 weeks of injections and continued to decrease over the course of 8 weeks (*p*<0.05) (Figure 1D). Compared to sham operated mice, both AKP-3 (duodenal IAP) and AKP-6 (global IAP) gene expression decreased in the duodenum of both the CBDL and CCl4 induced liver fibrosis models (Figure 1E-F).

### IAP regulates gut barrier in murine liver fibrosis models

**Figure 2:**
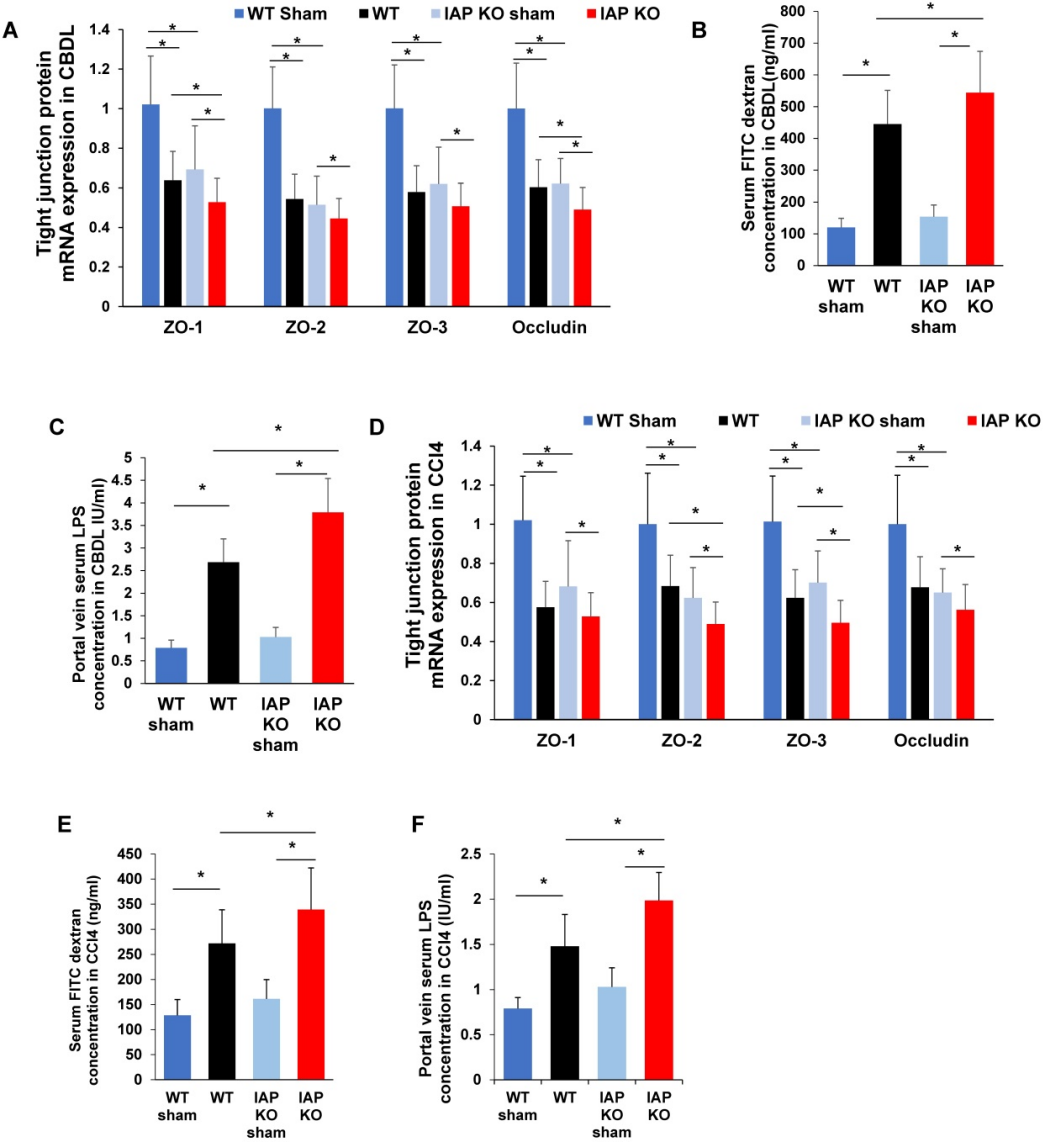
** IAP regulates intestinal tight junction protein expression in models of liver fibrosis in mice.** (A) Terminal ileum tight junction protein genes (ZO-1, ZO-2, ZO-3, Occludin) expression in sham or common bile duct ligated mice detected by qPCR in WT and IAP KO animals. (B) Serum FITC-dextran level 4-hour after gavage at indicated groups. (C) Serum LPS concentration measured by LAL assay. (D) Terminal ileum level of tight junction proteins (ZO-1, ZO-2, ZO-3, Occludin) expression after 8 weeks of CCl4 induced liver fibrosis determined by qPCR. Serum FITC-dextran (E) level 4-hour after gavage and serum LPS concentration (F) at indicated groups measured by LAL assay. *: *P*<0.05.

Impairment of the gut barrier is a critical component in the development of liver fibrosis. IAP has been shown to play a critical role in maintaining intestinal tight junction protein expression. We therefore tested the gut barrier function in CBDL and CCl4 injected WT and IAP KO mice. Terminal ileum mRNA expression of ZO family and Occludin were significantly decreased in WT and IAP KO mice after both CBDL and CCl4 injection. At 3 weeks post-CBDL, ZO-2, ZO-3 and Occludin decreased more in WT mice than in IAP KO mice (Figure 2A). At 8 weeks post CCl4 injection, ZO-1 and Occludin were more decreased in WT than in IAP KO mice (Figure 2D).

In both WT and KO mice, serum FITC dextran was significantly higher post-CBDL than in sham operated mice, indicating impaired gut barrier function. As expected, IAP KO mice had worse gut barrier function than WT mice as evidenced by higher serum FITC dextran levels (*p*<0.05) (Figure 2B). Terminal ileum ZO-1 and Occludin expression significantly decreased after 3 weeks of CBDL, with lower levels in IAP KO mice than WT mice (*p*<0.05) (Figure 2A). Portal vein serum LPS concentration was measured as the portal venous system represents the most proximal exit point for bacterial inflammatory mediators originating from the gut lumen, and is another marker of gut barrier function. Portal vein LPS was significantly higher in mice that underwent CBDL, and IAP KO mice had a significantly higher portal vein LPS concentration when compared to WT (Figure 2C). CCl4-treated mice had a similar pattern to CBDL mice. All CCl4 injected mice had significantly increased serum FITC dextran and portal vein LPS concentrations, as well as decreased ileum tight junction gene expression when compared to vehicle treated mice after 8 weeks (Figure 2E-F).

### Lack of IAP results in a severe CBDL and CCL4 induced-liver fibrosis in mice

**Figure 3:**
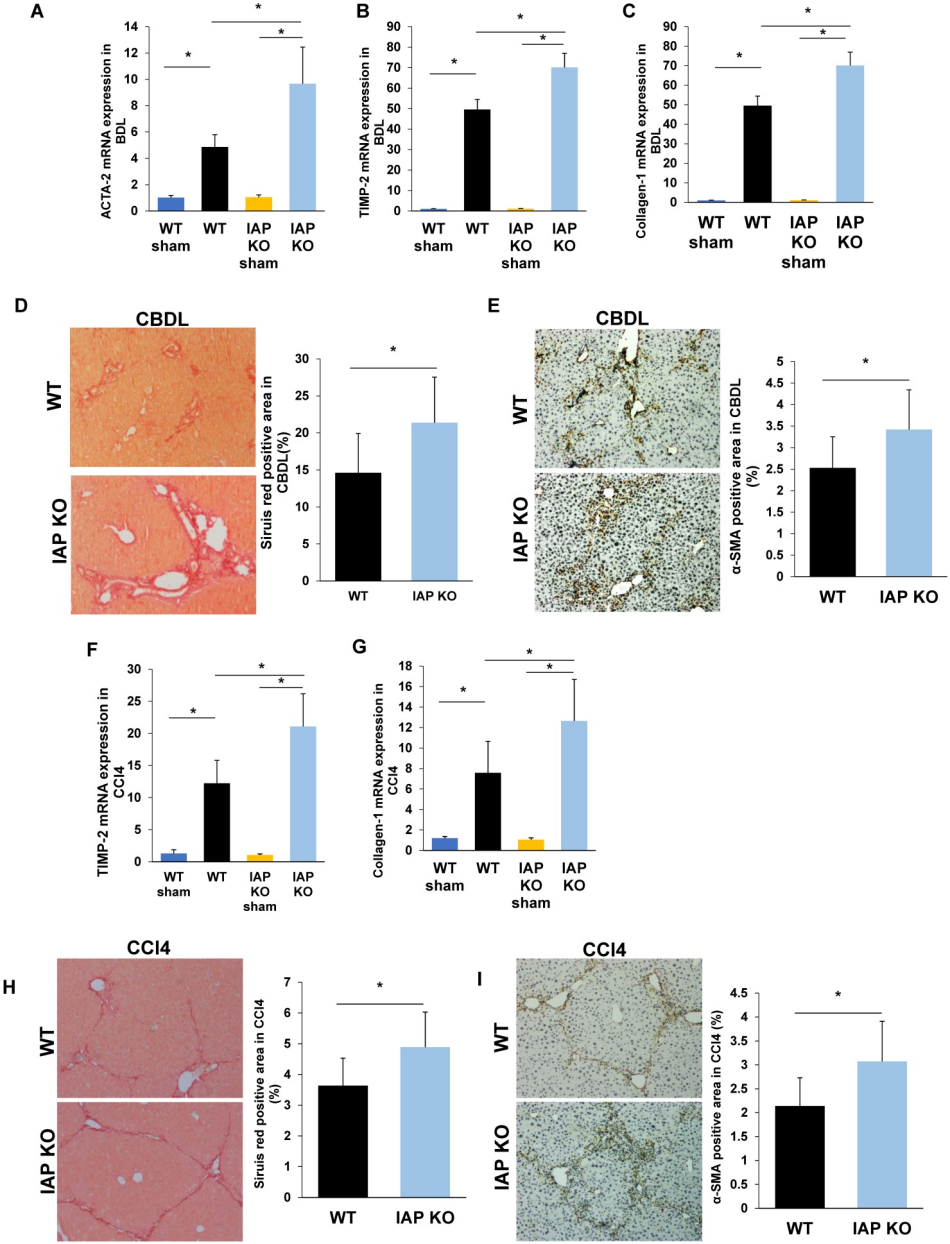
** Lack of IAP leads to a severe liver fibrosis after CBDL and CCL4 treatments.** (A-C) WT and IAP KO mice underwent CBDL for 3 weeks, liver levels of ACTA-2, TIMP-2 and Collagen-1 genes were determined by qPCR, and compared with WT mice; (D,E) 3 weeks of CBDL mice were tested for fibrillar collagen by Sirius red staining (×100) and expression of a-SMA was determined by immunohistochemistry (×200). (F-H) WT and IAP KO mice underwent CCl4 treatment for 8 weeks, liver levels of ACTA-2, TIMP-2 and Collagen-1 genes were determined by qPCR; I, J: 8 weeks of CCl4 injected mice were tested for fibrillar collagen by Sirius red staining (×100) and expression of a-SMA was determined by immunohistochemistry (×200). *: *P*<0.05.

As the gut lumen is a rich source of bacterial toxins, especially LPS, and these gut-derived mediators have been shown to participate in the pathogenesis of chronic liver inflammation/fibrosis 5, we hypothesized that the impaired gut barrier in IAP deficient mice may result in worsened liver fibrosis. We therefore examined liver fibrosis markers in both the CBDL and CCl4 models of liver injury and in both cases found a more severe damage and fibrosis with increased TIMP-2, Collagen-1, and ACTA-2 gene expressions. These genes associated with liver fibrosis also had higher expression in the IAP KO mice (Figure 3A-C, F-G). Sirius red staining of sham operated mice showed minimal staining of collagen fiber, while staining was prominent after both CBDL and CCl4 injections. Interestingly, collagen staining was more pronounced in the livers of IAP KO mice compared to WT mice (Figure 3D, 3H). Similarly, α-SMA, another marker of fibrosis that is typically only found in the smooth muscle cells of the intrahepatic blood vessels under normal circumstances, was significantly increased in the livers of IAP KO mice compared to WT mice in both CBDL and CCl4 models (Figure 3E, 3I).

### IAP supplementation protects the gut barrier in CBDL and CCL4 induced-liver fibrosis in mice

**Figure 4:**
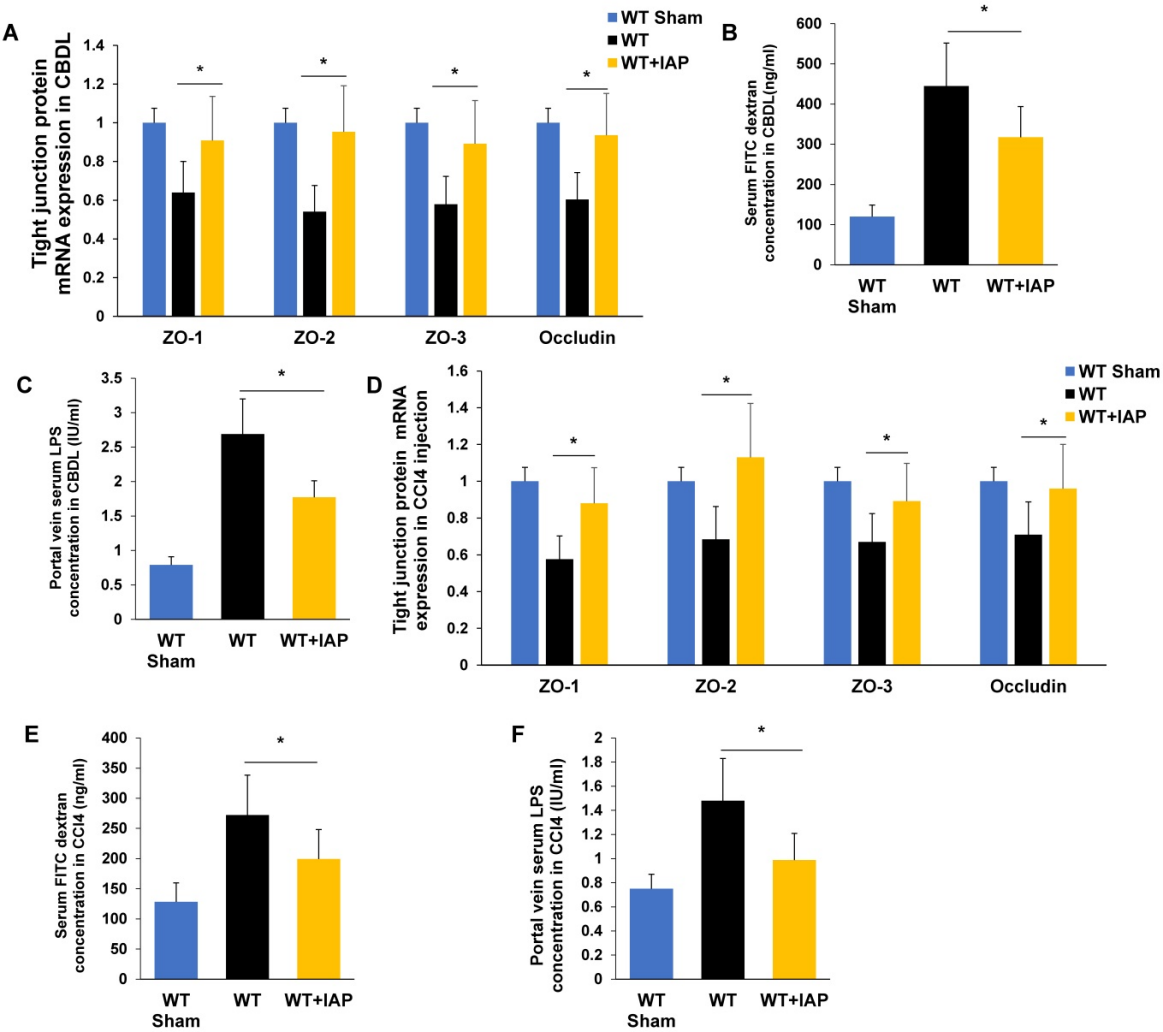
** IAP supplementation rescues the gut barrier in murine models of liver fibrosis.** (A) The level of ileal tight junction protein (ZO-1, ZO-2, ZO-3, Occludin) expression in mice treated with vehicle or IAP determined by qPCR, 3 weeks after CBDL. (B) Systemic serum FITC-dextran level 4-hour after oral gavage, and, (C) portal serum LPS concentration measured by LAL assay, 3 weeks after CBDL. (D) The level of tight junction protein (ZO-1, ZO-2, ZO-3, Occludin) expression was determined by qPCR, 8 weeks of CCl4 induced liver fibrosis. Systemic serum FITC dextran (E) and portal serum LPS concentration (F) after 8 weeks of CCl4 induced liver fibrosis. *:* P* <0.05.

We next tested whether supplementation with IAP in the drinking water could help to maintain the gut barrier function in murine models of liver fibrosis. As shown in Figure 4B and 4E, serum FITC labeled dextran concentrations increased in both WT mice and KO mice after CBDL and CCl4 injections, however, this increase was significantly attenuated by IAP supplementation. Furthermore, IAP supplementation also rescued the decrease in terminal ileum tight junction protein mRNA expression levels (Figure 4A, 4D). In addition, portal vein LPS concentration was also significantly decreased by IAP supplementation in both models. (Figure 4C, 4F).

### Oral IAP attenuates liver fibrosis in both CBDL and CCl4 models

**Figure 5:**
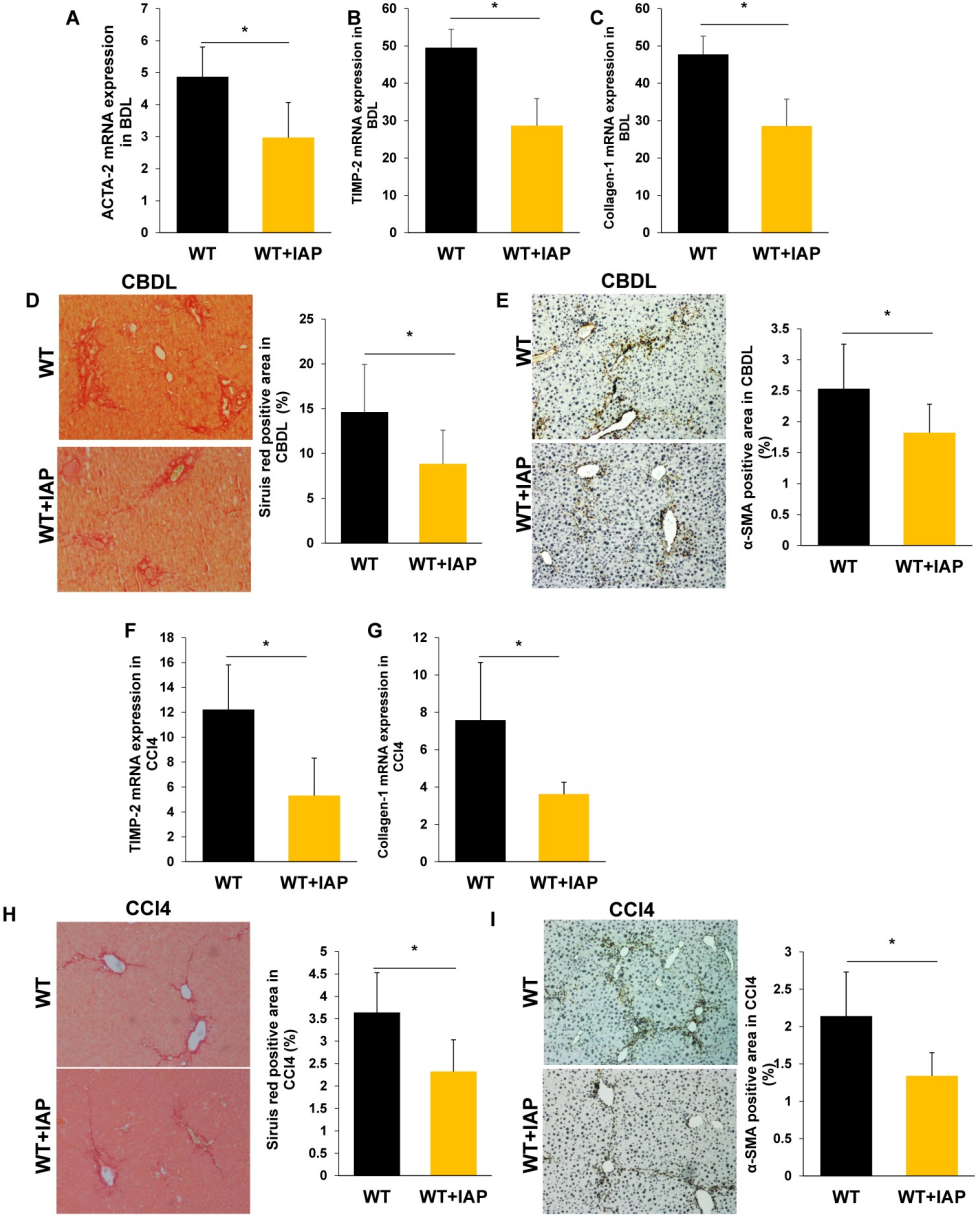
** Supplementation of IAP prevents the development of liver fibrosis in murine liver fibrosis models**. (A-C) liver levels of ACTA-2, TIMP-2 and Collagen-1 genes in WT mice treated with oral vehicle or IAP at 3 weeks after CBDL, determined by qPCR; (D,E) Liver fibrosis tested for fibrillar collagen by Sirius red staining (×100) and expression of a-SMA determined by immunohistochemistry (×200) mice underwent CBDL. (F-H) WT mice with supplementation of IAP underwent CCl4 treatment for 8 weeks, and levels of ACTA-2, TIMP-2 and Collagen-1 gene expression in the liver were determined by qPCR; (I, J) Mice treated with 8 weeks of CCl4 were tested for fibrillar collagen by Sirius red staining (×100) and expression of a-SMA was determined by immunohistochemistry (×200). *: *P*<0.05.

**Figure 6:**
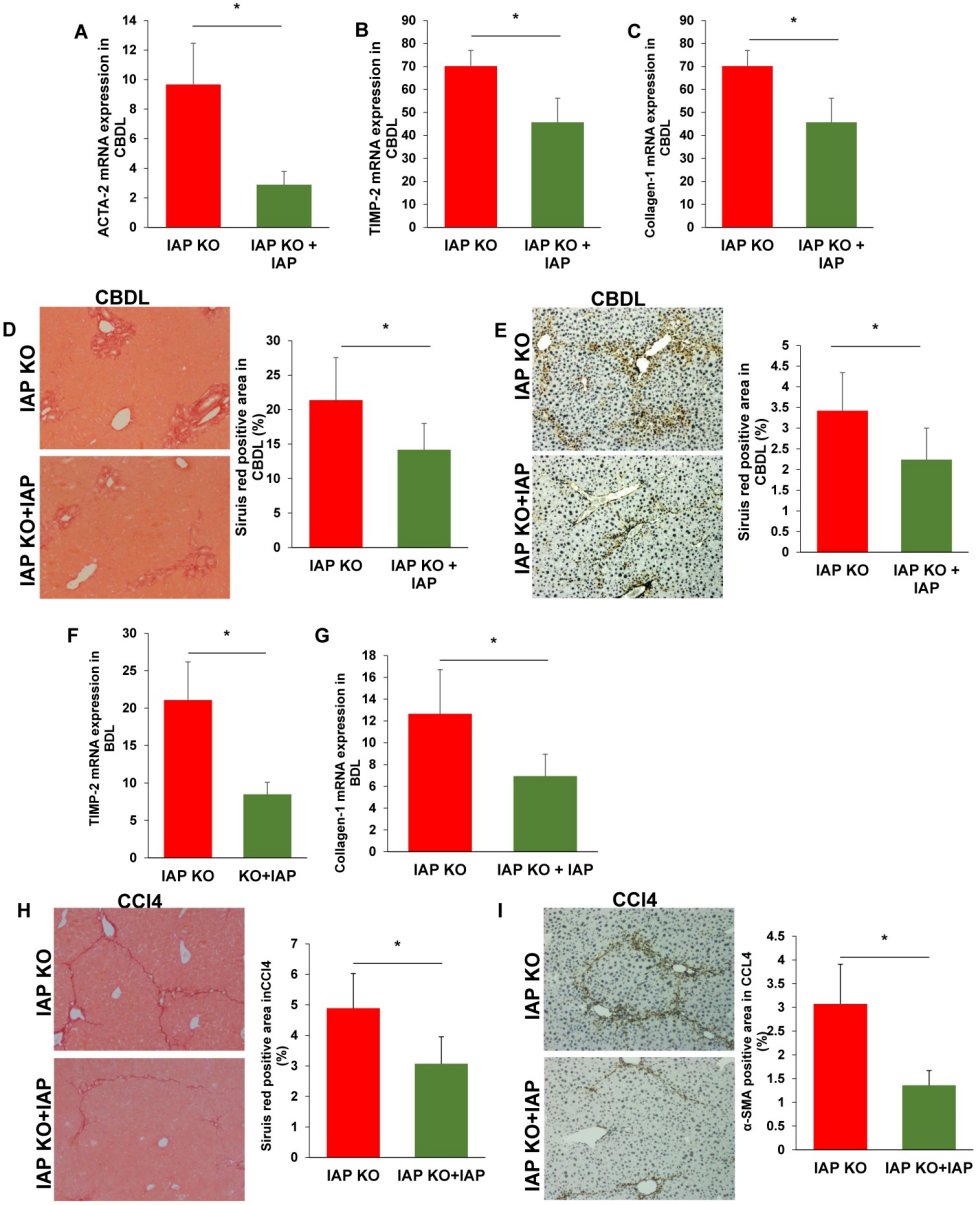
** Supplementation of IAP prevents the IAP KO mice development of liver fibrosis.** (A-C) IAP KO mice with oral supplement IAP and 3 weeks after CBDL, liver levels of ACTA-2, TIMP-2 and Collagen-1 genes were determined by qPCR; (D, E) Mice 3 weeks after CBDL were tested for fibrillar collagen by Sirius red staining (×100) and expression of a-SMA was determined by immunohistochemistry (×200). (F-H) IAP KO mice with supplementation of IAP underwent CCl4 treatment for 8 weeks, and levels of ACTA-2, TIMP-2 and Collagen-1 gene expression in the liver were determined by qPCR; (I, J) Mice treated with 8 weeks of CCl4 were tested for fibrillar collagen by Sirius red staining (×100) and expression of a-SMA was determined by immunohistochemistry (×200). *: *P*<0.05.

As translocation of gut derived LPS is a key driver of liver fibrosis, we hypothesized that oral IAP would maintain the gut barrier, detoxify luminal LPS, and attenuate the liver fibrosis induced by CBDL and CCl4 injury. After 3 weeks and 8 weeks of IAP supplementation in the CBDL and CCl4-treated mice respectively, our data suggested that oral IAP significantly decreased expression of TIMP-1, Collagen-1 and ACTA-2 in both WT and KO mice (Figure 5A-C, 5F-G; Figure 6A-C, 6F-G). On histologic evaluation, oral IAP resulted in a significant reduced Sirius red stained area for both WT and KO mice in both models compared to control WT and KO mice (Figure 5D, 5H; Figure 6D, 6H). Further, on immunohistochemistry, less staining of α-SMA was demonstrated in the livers of mice supplemented with IAP (Figure 5E, 5I; Figure 6E, 6I).

### IAP rescues CBDL and CCL4-induced liver fibrosis dependent of TLR4 pathway

**Figure 7:**
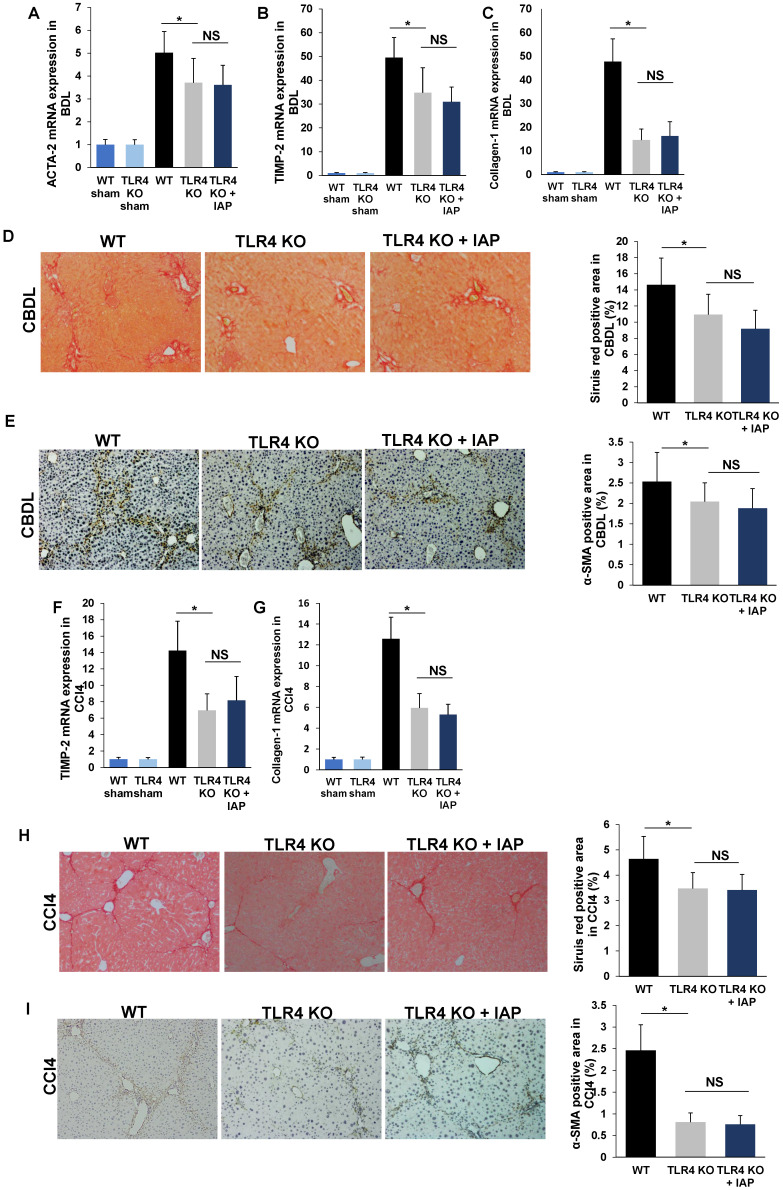
** IAP supplementation does not attenuate fibrosis in TLR4-KO mice**. (A-C) 3 weeks after CBDL in TLR4 KO mice, ACTA-2, TIMP-2 and Collagen-1 gene expression in the liver was compared from mice with and without IAP treatment. Sirius red staining (×100) and ɑ-SMA expression (×200) was also compared between the groups (D, E). The CCl4 induced liver fibrosis model was also used in TLR4 mice with IAP in drinking water. After 10 weeks, liver ACTA-2, TIMP-2 and Collagen-1 gene expression were detected by qPCR (F-H) and liver collagen fiber was detected by Siruis red staining (×100) (I), while ɑ-SMA was detected by immunochemistry method (×200) (J). *:* P* <0.05.

LPS induces liver fibrosis by translocating across the gut barrier, into the portal vein, where it then enters the liver, and activates the TLR4 pathway resulting in a variety of downstream signaling that lead to fibrosis 5. TLR4 KO mice have been shown to develop an attenuated degree of liver fibrosis in CBDL and CCl4 models compared to WT mice 5. In our study, mRNA expressions of ACTA-2, TIMP-2 and Collagen-1 were significantly lower in TLR4 mice compared to WT mice after both CBDL and CCl4 injection (Figure 7A-C, 7F-G). Sirius red and α-SMA positive staining area were also significantly decreased in TLR4 KO mice compared to WT mice (Figure 7D-E, 7H-I). This finding is consistent with previous results in both murine liver fibrosis models 5.

While it is known that LPS is a substrate for IAP, and that IAP decreases luminal LPS 6, we asked the question if the ability of IAP to prevent liver fibrosis is dependent on the TLR4 pathway. Accordingly, we performed our liver fibrosis models using TLR4 KO mice. We found that while TLR4 KO mice had lower TIMP-2, ACTA-2 and Collagen-1 mRNA expression than WT mice after CBDL, they did not display a significant difference between IAP supplementation and control-treated mice (Figure 7A-C,). Similarly, Sirius red staining and α-SMA immunochemistry staining also did not show a difference between IAP or vehicle-treated mice in CBDL models (Figure 7D-E). Similar results were shown in our CCl4 induced liver fibrosis model, as ACTA-2, TIMP-2 and Collagen-1 mRNA expression and Sirius red and α-SMA staining were similar between TLR4 KO and WT mice (Figure 7F-H, 7I-J).

## Discussion

CCl4 and CBDL are two of the most commonly studied liver fibrosis animal models. These models begin with an insult to the liver, by either direct damage to hepatocytes (CCl4) or intrahepatic cholestasis (CBDL), followed by induction of key inflammatory pathways. Ultimately, this leads to the secretion of inflammatory cytokines, which in turn activate hepatic stellate cells via intrahepatic pathways thereby driving the development of fibrosis 17. Importantly, the gastrointestinal tract and the liver are anatomically and physiologically connected by the portal venous system and biliary tree. An insult to the liver also manifests as changes to bile juice components and the gut microbiota, altering the gut epithelial barrier, further promoting translocation of pathogenic mediators into the portal venous system and liver 1. The effects of mucosal-driven metabolites and luminal microbes on liver are considered critical to the development of a variety of liver diseases 1. It has been well demonstrated that intestinal barrier dysfunction and increased gut permeability are drivers of liver cirrhosis 18, 19. In fact, gut barrier dysfunction not only occurs during the end stage of liver disease but likely begins to take place in its early stages. Hepatic insults like excessive alcohol intake and high-fat diet have been shown to induce gut barrier breakdown 2, 3. In murine models, expression of intestinal tight junction proteins is decreased as early as the first day after bile duct ligation or CCl4 injection 20. Therefore, in this study, we began IAP supplementation 4 days in advance of the initiation of the hepatic insults, cholestasis in the case of CBDL and hepatoxicity in the case of CCl4. Translocation of intestinal bacteria and their by-products induces liver inflammation and plays a critical role in liver fibrosis 5, 21. Breakdown of the protective gut barrier allows for more PAMPs such as LPS to pass through the intestine and become exposed to the liver. Despite the pathophysiologic importance of gut barrier alterations in the development of liver fibrosis, the mechanisms underlying the development of gut barrier dysfunction in this setting remain unclear.

IAP is well-known as a marker of enterocyte maturation and its activity has been negatively correlated with intestinal inflammation 6. IAP activity has been shown to be decreased in diseases of chronic intestinal inflammation like inflammatory bowel disease (IBD), alcohol consumption, celiac disease, metabolic syndrome, and obesity 7, 9, 14-16. Intestinal inflammation is also found in diseases such as nonalcoholic steatohepatitis (NASH), alcoholic liver disease (ALD), and other precursors to liver cirrhosis 22, 23. To our knowledge, this study is the first to demonstrate that IAP activity is decreased both in human patients with liver cirrhosis and in murine models of liver fibrosis. Given the decrease in IAP activity associated with liver fibrosis, we sought to explore its role in the pathophysiology of this condition.

While gut barrier function relies on many different factors, the central component of the intestinal barrier are enterocytes that are tightly bound to adjacent cells by apical junctional proteins that include claudins, occludins, E-​cadherins, desmosomes and junctional adhesion molecules 24, 25. The integrity of the intestinal barrier becomes impaired when any of its key components are lost or become dysfunctional. This dysfunction can be induced by physiologic insults leading to gut inflammation and/or intestinal dysbiosis, such as high fat diet, alcohol consumption, lack of bile acids, IBD, etc. 2, 3, 26. It has been shown before that IAP is essential for maintenance of the gut barrier in many disease models like systemic LPS exposure, fasting, alcohol-induced liver injury, and colitis 11, 27. When mice lack IAP, the gut barrier becomes significantly impaired and these mice are highly susceptible to physiologic insults that affect the gastrointestinal tract 11, 27. The present data adds to the myriad effects of IAP and suggest that gut barrier dysfunction is a critical step in development of liver fibrosis. When the gut barrier is damaged, intestinal bacteria-derived molecules, and even whole bacteria, will translocate from the gut to the liver via the portal vein, resulting in liver inflammation and injury, and ultimately liver fibrosis 5, 28. Further, when the liver becomes injured and cirrhosis develops, it also negatively impacts the gut barrier and increases bacteria translocation, leading to a vicious cycle of hepatic injury 29.

The key function of IAP is its ability to detoxify a variety of pro-inflammatory mediators that exist within the gut lumen, including LPS which is composed of hydrophilic polysaccharides within its core and O-antigen and a hydrophobic lipid A component. In recent years, LPS has been shown to be one of the key mediators linking the gut to the development of liver disease, as well as many other systematic diseases 30. IAP has been shown to remove a phosphate group from the lipid A moiety of LPS which leads to the amelioration of inflammatory activity of LPS 31-33. We have shown that IAP knockout mice have an impaired ability to detoxify luminal LPS and appear to be more susceptible to gut-derived inflammatory conditions 8, 15. This is likely exacerbated in the scenario of the vicious cycle between gut and liver injury. Supplemental IAP or recombinant alkaline phosphatase has been shown to prevent alcohol-induced hepatosteatosis and acute on chronic liver failure 9, 34. In this study, we provide evidencethat enteral supplementation of IAP in drinking water reduces the concentration of portal serum LPS that is derived from the gut lumen, thus implying that IAP may decrease the amount of active LPS that is translocated from the gut into the liver. This action likely further attenuates the vicious inflammatory cycle and the development of liver fibrosis. The fact that IAP supplementation was not able to attenuate liver fibrosis in TLR4 KO mice, confirms the important role of LPS in this setting.

It has been shown that targeting gram negative bacteria and LPS by long-term administration of rifaximin, polymyxin or norfloxacin may rescue liver fibrosis 28, 30, 35. However, chronic antibiotic therapy is inherently associated with many negative side effects. In another study, the probiotic amino acid glutamine was found to protect the gut barrier and alleviate liver fibrosis in murine models 36. The results from our study put forward another novel therapy that may potentially prevent liver disease from progressing to liver fibrosis and cirrhosis. Enteral IAP has been used in one human study with intraduodenal administration for 7 days in patients with severe Ulcerative Colitis 37. In this limited single arm trial, no safety issues, adverse events, or side effects were reported. Given that IAP is an endogenously produced enzyme, it is not surprising that it could be safely administered to patients with few if any detrimental effects 37. Although IAP is partially degraded in the stomach, oral IAP in the drinking water is a very easy route of administration and we have previously shown that this method of supplementation is effective to increase intestinal luminal concentration of IAP 12, 38. While these animal data are promising, whether IAP can be used to prevent humans from developing liver fibrosis will require a proper clinical trial.

Although IAP functions both to directly maintain the gut barrier and also to detoxify bacterial ligands such as LPS, it is not clear which function of IAP plays a more important role in its ability to attenuate liver fibrosis. We sought to address the mechanistic question by using the TLR4 KO mice. Our finding shows that the TLR4 KO mice did not have the same degree of improvement as wild type mice, suggests that this pathway is a critical one in regard to IAP action. With increased exposure of TLR4 to LPS and/or increased expression or sensitivity of TLR4, there is an inappropriate immune response which induces a chronic inflammatory liver disease 39, 40. In recent years, many studies have provided evidence supporting the role of the LPS/TLR4 pathway in nonalcoholic fatty liver disease, alcoholic liver disease, acute liver failure, chronic hepatitis B and C, primary sclerosing cholangitis, primary biliary cirrhosis, liver fibrosis, and even hepatocellular carcinoma 5, 41-47. In our experiments, IAP appears to rescue liver fibrosis only when TLR4 is present, suggesting that IAP protects the liver from fibrosis by potentially de-activating LPS, an important ligand in the TLR4 pathway.

In conclusion, our data show that IAP level decreased in liver fibrosis, likely contributing to a breakdown of the intestinal barrier. The gut barrier dysfunction results in an increased LPS translocation into the liver through the portal system, worsens liver damage, and creates a vicious cycle, and ultimately leads to liver fibrosis. We have shown that oral IAP could represent a novel therapy to prevent the development of liver fibrosis by detoxifying luminal LPS and protecting the gut barrier function, in a TLR4-dependent fashion.

## Supplementary Material

Supplementary material.

## Figures and Tables

**Table 1:** Characters for the patient with and without liver cirrhosis

	Cirrhosis				No-cirrhosis
	total	A class	B class	C calss	
Number	86	27	30	29	18
Male	51	16	16	19	10
Female	35	11	14	10	8
Hepatitis B	56	15	19	22	
Hepatitis C	15	5	6	4	
Alcoholic	6	3	2	1	
Autoimmune	5	3	1	1	
Cholestatic	4	1	1	2	
Unknown	3	1	2	0	
Smoke	24	11	6	7	4
Duration of disease (months)	18.81±25.87	14.74±21.04	16.55±21.37	24.65±32.65	
Total bilirubin (μmol/L)	53.70±54.82	23.56±8.17	34.23±27.13	101.90±67.88	14. 83±5.35
Scr (μmol/L)	67.94±34.30	63.59±23.26	62.07±32.75	78.06±42.88	55.89±18.81
Platelet (×109/L)	95.77±58.70	114.44±59.40	93.13±61.10	81.12±54.45	210.44±44.03
ALT (U/L)	146.37±137.82	58.78±44.27	114.47±69.14	260.93±160.72	20.22±10.73
AST (U/L)	146.92±128.48	62.30±39.51	120.97±75.56	252.55±153.51	25.61±10.06
Albumin (g/L)	34.05±8.92	41.11±4. 87	35.90±7.75	25.55±5.57	42.17±4.95
prothrombin time (s)	16.19±5.49	11.5±1.32	14.91±2.57	21.84±5.25	10.86±1.33
