# Supplementary material for: A role for intestinal alkaline phosphatase in preventing liver fibrosis
Source: Theranostics. 2021 Jan 1;11(1):14–26. doi: 10.7150/thno.48468 (PMC7681079; doi:10.7150/thno.48468)
Supplement: Supplementary file 1 — Supplementary material. [file thnov11p0014s1.pdf]

Bile duct ligation

Common bile duct ligation was performed as follows and 12 mice in each group. Male mice aged 10-11 weeks were anesthetized using isoflurane inhalation. The abdomen was clipped and prepared in sterile fashion using 70% ethanol. A longitudinal midline laparotomy incision about 2.5 cm was made and the common bile duct was dissected from the portal vein and then doubly ligated with 6-0 vicryl (Ethicon). The abdomen was then closed in two layers using running 5-0 vicryl suture. Sham operated animals underwent an identical procedure with the exception of the common bile duct ligation. Postoperatively, animals were resuscitated with 0.5 mL of normal saline injected subcutaneously and returned to clean cages with water and food *ad libitum*. Buprenorphine 0.24 mg/kg was injected subcutaneously every 12 hours for the first 48 hours after operation. All the mice were euthanized 3 weeks following the procedure.

CCl4 injection

CCl4 (Sigma) was dissolved in olive oil (Sigma) 1:4. Male mice (aged 7-8 weeks) were injected intraperitoneally with 2.5 μL/g twice per week (12 mice in each group). The mice were euthanized at 8 weeks, while TLR4 KO mice were euthanized at 10 weeks.

Serum LPS concentration determination

LPS concentration was detected by a commercial chromogenic LPS detection kit (Thermofisher) using the manufacturer's protocol. Briefly, serum samples diluted 1:50 (total volume 50 μL) were incubated with 50 μL LAL at 37 degrees for 10 minutes, followed by 100 μL of Chromogenic Substrate solution incubated at 37 degrees for 6 minutes. After adding 100 μL acetic acid, absorbance was measured at 410nm and LPS concentration was reported as IU/mL.

Immunochemistry

Fresh tissue was fixed in 4% paraformaldehyde solution overnight, dehydrated by ethanol, and then embedded in paraffin. Slices were cut at room temperature and deparaffinized with xylene substitution, and re-hydrated in ethanol. Antigens were unmasked by 10mM sodium citrate in a decloaking chamber and incubated at 0.5% H_2O_2 in methanol for 30 min, followed by cooling down to room temperature. Tissue was blocked in normal goat serum (DakoCytomation) diluted 1:3 and incubated with primary antibody overnight at 4 degrees Celsius. Cuts were then incubated with secondary antibody for 1 hour at room

temperature and washed with PBS. Slides were developed with ABC (Vector lab) DAB. The reaction was stopped with deionized water and then slides were counterstained with hematoxylin for 6min. Lastly, slides were dehydrated in ethanol followed by xylene substitute, then mounted.

qPCR

Tissues were homogenized in Trizol solution (Thermo Fisher), and RNA was extracted per the manufacturer's protocol. Extracted RNA was then treated with DNAse with Turbo DNA free kit (Thermofisher) per manufacturer's protocol. 1 μg RNA was used to make cDNA with iScript™ Reverse Transcription Supermix (Bio rad). The final qPCR reaction system was comprised of 2 μL cDNA, primers, water and iQ™ SYBR Green Supermix to a final volume of 20 μL. Expression of target gene mRNA was compared with GAPDH, and ΔΔCt method was used to provide relative quantity of gene expression. The average expression of the target gene in the control group was set as 1.

Intestinal Permeability evaluated by FITC dextran

Intestinal permeability was measured using low molecular weight FITC labeled dextran, which permeates across an injured epithelial barrier. 4-7 kD FITC-dextran was dissolved to 75 mg/mL and administered by oral gavage to mice at dose of 300 mg/kg 4 hours prior to sacrifice. Blood was obtained via cardiac puncture after euthanasia, allowed to clot for 30 minutes at room temperature, and then centrifuged to obtain serum. 25 μL of serum was then diluted by equal volume of PBS and serum FITC was determined by measuring excitation at 485nm and emission at 528nm. Serum FITC is reported as ng/mL of serum.
